# Supplementary material for: The interactome of intact mitochondria by cross-linking mass spectrometry provides evidence for coexisting respiratory supercomplexes
Source: Mol Cell Proteomics. 2017 Dec 8;17(2):216–32. doi: 10.1074/mcp.RA117.000470 (PMC5795388; doi:10.1074/mcp.RA117.000470)
Supplement: Supplemental Data [file supp_RA117.000470_133922_0_supp_23978_fzffwf.zip › spectra_annotation/mito_spectra_annotation/192-1-29-1-18-1.pdf]

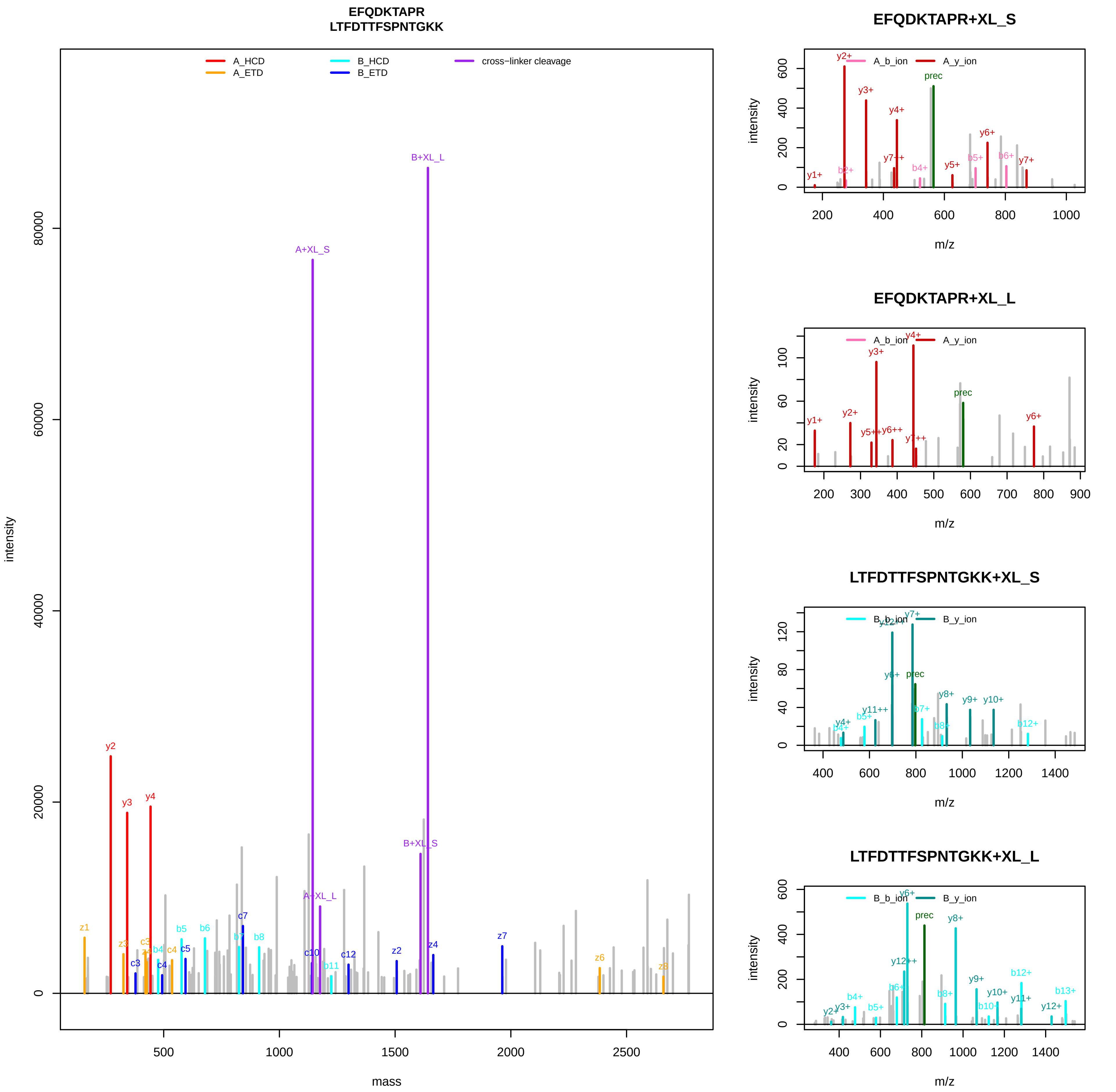

EFQDKTAPR
LTFDTTFSPNTGKK
A_HCD
A_ETD
B_HCD
B_ETD
cross−linker cleavage
B+XL_L
A+XL_S
B+XL_S
A+XL_L
y2
y3
y4
z1
z3
c3
z4
b4
c4
c5
b5
b6
b7
c7
b8
c10
b11
c12
z2
z4
z7
z6
z8
0
20000
40000
60000
80000
intensity
500
1000
1500
2000
2500
mass
EFQDKTAPR+XL_S
A_b_ion
A_y_ion
y1+
y2+
b2+
y3+
y4+
y7++
b4+
prec
y5+
b5+
y6+
b6+
y7+
0
200
400
600
200
400
600
800
1000
m/z
EFQDKTAPR+XL_L
y1+
y2+
y3+
y4+
y5++
y6++
y7++
prec
y6+
0
20
60
100
200
300
400
500
600
700
800
900
m/z
LTFDTTFSPNTGKK+XL_S
B_b_ion
B_y_ion
y7+
y12++
y6+
prec
y8+
y9+
y10+
y4+
b4+
b5+
y11++
b7+
b8+
b12+
0
40
80
120
400
600
800
1000
1200
1400
m/z
LTFDTTFSPNTGKK+XL_L
y6+
prec
y8+
y12++
b6+
b4+
y2+
y3+
b5+
b8+
y9+
y10+
b10+
y11+
b12+
b13+
y12+
0
200
400
600
400
600
800
1000
1200
1400
m/z
